# Reduced specialized processing in psychotic disorder: a graph theoretical analysis of cerebral functional connectivity

**DOI:** 10.1002/brb3.508

**Published:** 2016-06-29

**Authors:** Sanne C. T. Peeters, Ed H. B. M. Gronenschild, Therese van Amelsvoort, Jim van Os, Machteld Marcelis, Genetic Risk and Outcome of Psychosis (G.R.O.U.P.), Rene Kahn, Durk Wiersma, Richard Bruggeman, Wiepke Cahn, Lieuwe de Haan, Carin Meijer, Inez Myin‐Germeys

**Affiliations:** ^1^ Department of Psychiatry & NeuropsychologySchool for Mental Health and NeuroscienceEURONMaastricht University Medical CenterPO Box 6166200MD MaastrichtThe Netherlands; ^2^ Faculty of Psychology and Educational SciencesOpen University of the NetherlandsHeerlenThe Netherlands; ^3^ Department of Psychosis Studies Institute of PsychiatryKing's Health PartnersKing's College LondonLondonUK; ^4^ Institute for Mental Health Care Eindhoven (GGzE)EindhovenThe Netherlands

**Keywords:** Brain connectomics, functional magnetic resonance imaging, graph theory, psychotic disorders, siblings

## Abstract

**Background:**

Previous research has shown that the human brain can be represented as a complex functional network that is characterized by specific topological properties, such as clustering coefficient, characteristic path length, and global/local efficiency. Patients with psychotic disorder may have alterations in these properties with respect to controls, indicating altered efficiency of network organization. This study examined graph theoretical changes in relation to differential genetic risk for the disorder and aimed to identify clinical correlates.

**Methods:**

Anatomical and resting‐state MRI brain scans were obtained from 73 patients with psychotic disorder, 83 unaffected siblings, and 72 controls. Topological measures (i.e., clustering coefficient, characteristic path length, and small‐worldness) were used as dependent variables in a multilevel random regression analysis to investigate group differences. In addition, associations with (subclinical) psychotic/cognitive symptoms were examined.

**Results:**

Patients had a significantly lower clustering coefficient compared to siblings and controls, with no difference between the latter groups. No group differences were observed for characteristic path length and small‐worldness. None of the topological properties were associated with (sub)clinical psychotic and cognitive symptoms.

**Conclusions:**

The reduced ability for specialized processing (reflected by a lower clustering coefficient) within highly interconnected brain regions observed in the patient group may indicate state‐related network alterations. There was no evidence for an intermediate phenotype and no evidence for psychopathology‐related alterations.

## Introduction

The human brain as a complex system has been analyzed extensively using functional MRI (fMRI). At present, resting‐state fMRI research has predominantly focused on functional connectivity, that is, temporal correlation between spatially distinct regions, in specific brain networks (Friston and Frith 1995; Rotarska‐Jagiela et al. 2010; Ma et al. 2012). Studies have shown that the spontaneous, low frequency (0.1 Hz) fluctuations of the blood‐oxygenation‐level‐dependent (BOLD) signal measured in the absence of a goal‐directed task, show a high degree of coherence and spatial organization and correspond to functionally relevant resting‐state networks (Fornito et al. 2010). Therefore, it has been postulated that the resting state represents an intrinsic property of the functional brain organization.

There are two general methods to measure resting‐state functional connectivity: (1) seed‐based correlation analysis, which is hypothesis‐driven; and (2) independent component analysis (ICA), which is a multivariate, model‐free, data‐driven method (Karbasforoushan and Woodward 2012). More recently, another method for analyzing resting‐state fMRI at a whole brain level has been introduced, called graph theory. In graph theory, the human brain is described and analyzed as a graph (network) with brain regions as graph nodes and the functional connection between nodes as graph edges (Bassett and Bullmore 2006; Bullmore and Sporns 2009; Newman 2010; Fan et al. 2011); also known and for the first time described as the human connectome (Sporns et al. 2005). Studies using graph analysis have shown that the human brain demonstrates small‐world properties, meaning that the network has a highly clustered local connectivity (greater than in a random network) and that there is a shorter path length (in terms of shortest distance) between brain regions than would be expected in a regular network (Watts and Strogatz 1998; Achard et al. 2006). Small‐world networks support both local specialization and global integration, and confer resilience against pathological influences (Achard and Bullmore 2007), but also maximize the efficiency of information processing at a low wiring cost (Ding et al. 2011). This small‐world construction is noticeable in both structural and functional brain networks at the whole brain level (Ding et al. 2011; Alexander‐Bloch et al. 2013a).

Recently, graph theoretical methods have been applied to better understand the brain and its dysconnectivity in psychiatric disorders, such as schizophrenia (Karbasforoushan and Woodward 2012). These few studies have shown that patients with schizophrenia have alterations in diverse topological properties of the functional human brain network with respect to controls (Liu et al. 2008; Bullmore and Sporns 2009; Alexander‐Bloch et al. 2010; Ma et al. 2012). That is, functional networks of patients with schizophrenia have been characterized by reduced small‐worldness, and reduced clustering coefficient and local efficiency (i.e., reduced specialized local information processing) (Liu et al. 2008; Alexander‐Bloch et al. 2010; Lynall et al. 2010; Yu et al. 2011; Fornito et al. 2012). In addition, a longer characteristic path length and lower global efficiency (i.e., reduced ability to specialized parallel information processing between dispersed brain regions) have been found in schizophrenia (Liu et al. 2008; Yu et al. 2011) but there are also reports of shorter path length and higher global efficiency (Alexander‐Bloch et al. 2010; Lynall et al. 2010). In other words, the evidence to date suggests that functional brain networks have a more random organization in patients with schizophrenia than in healthy controls. There are not many family or twin studies with a graph theoretical network design. A study in healthy monozygotic and dizygotic twins has shown that brain network functional connectivity has heritable cost‐efficient properties (Fornito et al. 2011). In addition, a graph theoretical network study of patients with schizophrenia and first‐degree relatives of patients has shown similar functional network randomization between these two groups, suggesting that this represents a marker of familial risk (Lo et al. 2015). The thus far more frequently used traditional methods (seed‐based correlation, ICA) consistently showed that unaffected siblings/first‐degree relatives share functional connectivity network alterations with their affected siblings (e.g., Whitfield‐Gabrieli and Ford 2012; Fornito et al. 2013; Su et al. 2013), with only a few exceptions, for example, (Repovs et al. 2011; Khadka et al. 2013). Furthermore, insight into the role of functional brain network may yield (intermediate) phenotypes derived from clinical‐behavioral correlations of these topological measures. Previous research suggests that the complex clinical presentations (psychotic symptoms and cognitive alterations) of schizophrenia may be related to abnormal integration between spatially distinct brain areas and inefficient information processing (Bullmore et al. 1997; Friston 1998; Stephan et al. 2006; Wang et al. 2012) which, from a graph theoretical network perspective, would be supported by reduced clustering coefficient, reduced local efficiency, longer path length, and lower global efficiency. To date, only few studies investigated the association between topological organization and symptoms of schizophrenia (Liu et al. 2008; Lynall et al. 2010; Yu et al. 2011). Two of these studies did not find an association between topological measures and clinical correlates, but Yu et al. (2011) reported that negative symptoms were associated with a longer characteristic path length and lower global efficiency, whereas no association with positive symptoms was found.

Following from the above, we hypothesized that patients with psychotic disorder and unaffected siblings would reveal abnormalities in topological properties of brain network connectivity (i.e., reduced small‐worldness, reduced clustering coefficient, and increased path length). In addition, exploratory analyses were performed to investigate the associations between topological measures and symptomatology (i.e., positive and negative symptoms, disorganization, excitement, emotional distress, as well as neuro‐ and social cognition).

## Methods

### Participants

Data pertain to baseline measurements of a longitudinal MRI study in Maastricht, the Netherlands. For recruitment and inclusion criteria of patients, their siblings and healthy controls, see Habets et al. (2011). Diagnosis was based on the Diagnostic and Statistical Manual of Mental Disorder‐IV (DSM‐IV) criteria (American Psychiatric Association, 2000), assessed with the Comprehensive Assessment of Symptoms and History (CASH) interview (Andreasen et al. 1992). The CASH was also used to confirm the absence of a diagnosis of nonaffective psychosis in the siblings and absence of a lifetime diagnosis of any psychotic disorder or current affective disorder in the healthy controls. The occurrence of any psychotic disorder in first‐degree family members also constituted an exclusion criterion for the controls. Before MRI acquisition, participants were screened for the following exclusion criteria: (1) brain injury with unconsciousness of >1 h, (2) meningitis or other neurological diseases that might have affected brain structure or function, (3) cardiac arrhythmia requiring medical treatment, and (4) severe claustrophobia. In addition, participants with metal corpora aliena were excluded from the study, as were women with an intrauterine device and (suspected) pregnancy.

The sample comprised 73 patients with psychotic disorder, 83 siblings of patients with psychotic disorder, and 72 controls. Forty‐six families participated: 25 families with one patient and one sibling, three families with one patient and two siblings. One family with two patients, six families with two siblings, and two families with one patient and three siblings. In the control group, there were nine families with two siblings. In addition, 41 independent patients, 34 independent siblings, and 54 independent controls were included.

Patients were diagnosed with: schizophrenia (*n* = 47), schizoaffective disorder (*n* = 9), schizophreniform disorder (*n* = 4), brief psychotic disorder (*n* = 2), and psychotic disorder not otherwise specified (*n* = 11). Ten controls and 16 siblings were diagnosed (lifetime) with major depressive disorder, but none of them presented in a current depressive state.

The standing ethics committee approved the study, and all the subjects gave written informed consent in accordance with the committee's guidelines and with the Declaration of Helsinki (Nylenna and Riis 1991).

### Clinical assessment

The positive and negative syndrome scale (PANSS) (Kay et al. 1987) was used to measure recent symptomatology. The Five Factor Model by van der Gaag (2006) was used, dividing the PANSS in Positive symptoms, Negative symptoms, Disorganization symptoms, Excitement, and Emotional Distress (van der Gaag et al. 2006). Siblings and healthy controls were assessed with the Structured Interview for Schizotypy‐revised (SIS‐R) (Vollema and Ormel 2000) to assess schizotypy.

Educational level was defined as highest accomplished level of education. Handedness was assessed using the Annett Handedness Scale (Annett 1970).

### Neuropsychological assessment

Attention/vigilance was assessed using a Continuous Performance Test (CPT‐HQ) with working memory (WM) load, also known as CPT‐AX (Nuechterlein and Dawson 1984) (longer reaction times reflecting worse performance). The Wechsler Adult Intelligence Scale (WAIS)‐III (Wechsler 1997) subtest Arithmetic was used to measure WM, which addresses both verbal comprehension and arithmetic skills. Two areas of social cognition that have been associated with psychotic symptoms were investigated, that is, facial emotion processing and theory of mind (ToM) (Penn et al. 2008; de Achaval et al. 2010). Facial emotion processing was measured with the Degraded Facial Affect Recognition task (DFAR) using the overall proportion of correct answers (van ‘t Wout et al. 2004), whereas ToM was assessed using the raw scores of the hinting task (Versmissen et al. 2008). The hinting task assesses the mentalizing‐capacity required to comprehend real intentions behind indirect speech. For the Arithmetic, DFAR and hinting task, higher scores indicate better performance.

### Substance use

Substance use was measured with the Composite International Diagnostic Interview (CIDI) sections B, J, and L (WHO, 1990). Use of cannabis and other drugs was based on the lifetime number of instances of drug use. CIDI frequency data on lifetime cannabis use were available for 220 participants (4% missing data). Data on other drug use were available for 223 participants (2% missing data). Data on cigarette smoking and alcohol use were available for 212 participants (7% missing data) and 206 participants (9% missing data), respectively.

### Antipsychotic medication use

In the patient group, antipsychotic medication use was determined by patient reports and verified with the treating consultant psychiatrist. Best estimate lifetime (cumulative) antipsychotics (AP) use was determined by multiplying the number of days of AP use with the corresponding haloperidol equivalents and summing these scores for all periods of AP use (including the exposure period between baseline assessment for the G.R.O.U.P. study and the moment of baseline MRI scanning), using the recently published conversion formulas for AP dose equivalents described in Andreasen et al. (2010).

### MRI acquisition

Functional and anatomical MRI images were acquired using a 3T Siemens Magnetom Allegra head scanner (Siemens Medical System, Erlangen, Germany). The functional resting state data were acquired using an echo‐planar imaging (EPI) sequence: number of volumes: 200; echo time (TE): 30 msec; repetition time (TR): 1500 msec; voxel size: 3.5 × 3.5 × 4 mm^3^; flip angle 90°; total acquisition time: 5 min. During the scan, participants were instructed to lie with their eyes closed, think of nothing in particular, and not fall asleep. In addition, anatomical MRI scans had the following acquisition parameters: (1) Modified Driven Equilibrium Fourier Transform (MDEFT) sequence: number of slices: 176; voxel size: 1 mm isotropic; TE: 2.4 msec; TR: 7.92 msec; inversion time: 910 msec; flip angle: 15°; total acquisition time: 12 min 51 sec; (2) Magnetization Prepared Rapid Acquisition Gradient‐Echo (MPRAGE; Alzheimer's Disease Neuroimaging Initiative) sequence: number of slices: 192; voxel size: 1 mm isotropic; TE: 2.6 msec; TR: 2250 msec; inversion time: 900 msec; flip angle 9°, total acquisition time: 7 min 23 sec. The matrix size was 256 × 256 and field of view was 256 × 256 mm^2^. Two sequences were used because of a scanner update during data collection. The MPRAGE and MDEFT are very similar, but to prevent systematic bias, the total proportion of MPRAGE scans (44%) was balanced between the groups.

### Data preprocessing and analysis

Image preprocessing was carried out on a Macintosh using the fMRI Signal Processing Toolbox (SPT v1.1), University of Cambridge as described in Jo et al. (2013) and Patel et al. (2014). The first four volumes of each resting state data set were removed to eliminate the nonequilibrium effects of magnetization. Preprocessing steps included slice‐time correction, temporal despiking, temporal bandpass filtering (0.02–0.1 Hz), coregistration to structural scan, spatial normalization, and spatial smoothing (6‐mm full‐width at half‐maximum Gaussian kernel). This toolbox corrects for motion by regressing out motion parameters, their first temporal derivatives, and cerebrospinal fluid (CSF) signal from ventricular regions.

**Figure 1:**
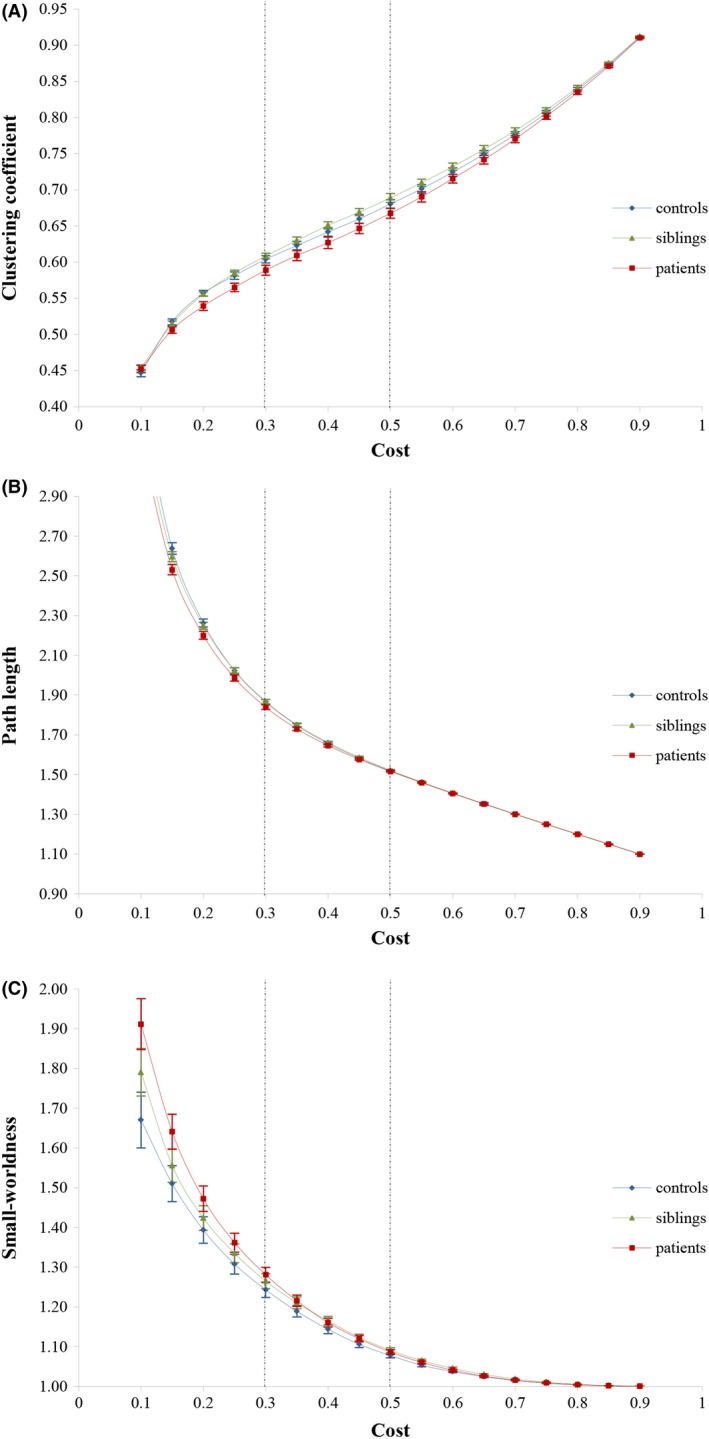
Topological measures of each group. Mean clustering coefficient (A), characteristic path length (B) and small‐worldness (C) for patients with psychotic disorder (red squares), siblings of patients with psychotic disorder (green triangles) and controls (blue diamonds) as a function of cost. Error bars correspond to standard error of the mean. The dotted lines represent the cost range (i.e., 0.30–0.50) that was used to calculate the mean of each topological measure.

The fMRI data were segmented into 90 regions (45 for each hemisphere) using the anatomically labeled template (AAL) reported by Tzourio‐Mazoyer et al. (2002). Regional mean time series over all voxels in each of the regions were computed and constituted the set of regional mean time series used for Pearson correlation analysis. Functional connectivity was then estimated by calculating the correlation between the mean time series of each pair of brain regions for each subject. A Fisher's *r*‐to‐*z* transformation was used on the Pearson correlation matrix in order to improve the normality of the Pearson correlation coefficients. Binary graphs were constructed by thresholding each subject's correlation matrix using a minimum spanning tree (MatLab BGL toolbox, http://dgleich.github.io/matlab-bgl/) followed by global thresholding (Alexander‐Bloch et al. 2010). In this sense, edges represented the correlations that were greater than the threshold, whereas no edges existed when the threshold was not surpassed. Graphs were constructed over a range of network costs, ranging from 0.1 to 0.9 at intervals of 0.05. The network cost refers to the number of edges in proportion to all possible edges included in the graph, such that at a cost equal to one there would be edges from each node to every other node (Alexander‐Bloch et al. 2013b). Group differences on topological properties were measured using a summary statistic following Alexander‐Bloch et al. (2010), that is, the mean of each topological measure was calculated over the range of costs from 0.3 to 0.5 (Fig. 1). Reasons to choose this range were: (1) previous work suggests that above a cost of 0.5 graphs become more random (Humphries et al. 2006), and less small‐world; and (2) topological measures are rather constant over this range (Alexander‐Bloch et al. 2010).

Topological properties of the brain functional network were calculated with the Brainnetome toolkit (BRAT: http://www.brainnetome.org/brat). The topological measures that were used for group analyses included the (1) clustering coefficient (extent of the local density or cliquishness of a network), (2) shortest characteristic path length, and (3) small‐world properties (Liu et al. 2008; Rubinov and Sporns 2010) (see Data S1 for detailed description of these measures).

### Statistical analyses

For every participant, the topological measures (i.e., clustering coefficient, shortest characteristic path length, and small‐world properties) were exported to STATA version 12 (StataCorp., 2011).

Group differences in topological measures were analyzed by fitting multilevel random regression models (Goldstein 1987) given hierarchical clustering, occasioned by the fact that participants were clustered in families, compromising statistical independence of the observations. This was done using the XTREG command in STATA (StataCorp 2011). Topological measures were the dependent variables and group was the independent variable. Group was entered as linear and dummy variables (controls = 0, siblings = 1, patients = 2). Analyses were adjusted for the a‐priori hypothesized confounders: age, sex, handedness, and level of education. In separate analyses, correction for the additional confounders tobacco, alcohol, cannabis, and other drugs was applied. Although not previously investigated, these confounders may affect the topological measures since studies have shown that these substances have an influence on functional connectivity (Volkow et al. 2008; Roberts and Garavan 2010; Tomasi et al. 2010; Ma et al. 2011; Ding and Lee 2013). The patient population included 26 patients with a diagnosis other than schizophrenia. Planned sensitivity analyses were conducted by excluding these individuals from the analyses. Furthermore, to examine whether participants of the combined sibling and control group with higher schizotypy scores would be more similar to patients with respect to the topological outcome measures, analyses were repeated with a low and high schizotypy group (based on the median score). Schizotypy was based on SIS‐r mean scores on referential thinking, suspiciousness, magical ideation, illusions, psychotic phenomena, derealization/depersonalization, social isolation, introversion, sensitivity, restricted affect, disturbances in associative and goal‐directed thinking, poverty of speech, and eccentric behavior were entered in the analyses.

Associations between topological measures (independent variable) and (subclinical) psychotic symptoms/(social) cognitive performance (dependent variable) were examined. In patients, the association between three topological measures and five PANSS psychotic symptoms (positive, negative, disorganized, excitement, and emotional distress) was corrected for age, sex, lifetime AP exposure, and illness duration. In siblings and controls the association between topological measures and subclinical psychotic symptoms (SIS‐r) was corrected for group, age, and sex.

Associations with (social) cognitive performance were investigated in the combined group (of patients, siblings, and controls). To examine whether the association between topological measures and (social) cognitive performance (dependent variable) would be conditional on group, interactions were tested between group and three topological measures. In case of significant interactions, stratified effect sizes for the topological measures were calculated for each group using the Stata MARGINS routine. Analyses with (social) cognitive performance were corrected for group, age, sex, handedness, and educational level. Since these analyses were exploratory in nature, in order to generate hypotheses, we used a statistical significance level of *P *<* *0.05 (uncorrected).

Associations between AP medication and topological measures were analyzed in patients, with AP medication as independent variable and age, sex, and illness duration as confounders.

To control for type I error, significant *P*‐values were subjected to correction for multiple testing using the Simes method (Simes 1986). The Simes method avoids overcorrection associated with the Bonferroni correction if the statistical tests are not independent, as was the case in this study.

## Results

### Descriptive analyses

Table 1 shows the characteristics of the three groups. There were more men than women in the patient group, whereas the opposite held for the control group. Patients had lower educational level than controls and siblings. The study comprised a relatively stable patient group as reflected by the low PANSS scores. Patients had lower scores on the arithmetic and hinting task compared to controls and siblings, indicating worse WM and ToM. Patients had a longer reaction time on the CPT‐HQ compared to controls, reflecting a worse span of attention/vigilance. No differences in performance were observed for the DFAR task (emotion processing).

**Table 1:** Demographic characteristics of participants

	Patients (*N* = 73)	Siblings (*N* = 83)	Controls (*N* = 72)
Age at scan	27.8 (6.6)	29.6 (9.1)	30.0 (10.8)
Sex *n* (%) male	49 (65%)	45 (54%)	26 (36%)
Handedness	72.1 (63.9)	80.1 (53.8)	73.5 (61.2)
Level of education	4.2 (2.0)	5.2 (1.9)	5.4 (1.8)
PANSS positive	9.7 (4.1)	7.4 (1.5)	7.3 (1.2)
PANSS negative	11.9 (6.1)	8.5 (2.2)	8.2 (1.0)
PANSS disorganization	12.0 (3.3)	10.4 (1.0)	10.2 (1.2)
PANSS excitement	9.9 (2.9)	8.6 (1.4)	8.3 (1.1)
PANSS emotional distress	12.7 (5.2)	9.9 (2.7)	9.3 (2.1)
SIS‐r‐positive subscale		0.6 (0.4)	0.5 (0.5)
SIS‐r‐negative subscale		0.3 (0.3)	0.3 (0.2)
WAIS‐III arithmetic	12.5 (4.2)	15.3 (3.7)	15.5 (4.1)
CPT‐HQ reaction time	442.3 (91.8)	414.9 (76.6)	412.3 (82.7)
DFAR	71.2 (10.4)	71.8 (8.4)	73.0 (8.6)
Hinting task	18.0 (2.9)	19.2 (1.3)	19.3 (1.1)
Cannabis use^a^	37.2 (99.5)	6.7 (41.4)	6.0 (43.8)
Other drug use^b^	21.2 (68.3)	0.5 (4.4)	5.2 (43.5)
Cigarettes use^c^	11.4 (11.0)	2.6 (6.2)	1.9 (6.1)
Alcohol use^d^	6.7 (13.0)	10.1 (17.7)	5.1 (7.2)
Age of onset (years)	21.4 (6.8)		
Illness duration (years)	6.4 (3.7)		
Lifetime exposure to AP^a^	7022.9 (6711.3)		

SD, standard deviation; PANSS, positive and negative syndrome scale; SIS‐r, Structured Interview for Schizotypy‐revised; WAIS, Wechsler Adult Intelligence Scale; CPT‐HQ, continuous performance test; DFAR, degraded facial affect recognition; AP, antipsychotics.

Means (SDs) are reported.

a Number of times past year.

b Number of times past year.

c Average number of daily consumptions over the last 12 months.

d Average number of weekly consumptions over the last 12 months.

Cumulative exposure to AP medication, expressed in haloperidol equivalents.

Out of 73 patients, 64 used AP medication at the time of scanning (second generation: *n* = 60; first generation: *n* = 4). The mean current dosage of AP medication in terms of standard haloperidol equivalents was 5.3 mg (SD = 4.8 mg). Furthermore, 12 patients used antidepressants, three used benzodiazepines, five used anticonvulsants, and one used lithium. Two siblings and two controls used antidepressants, and one control used benzodiazepines.

### Associations between group and topological measures

Figure 1 supports the abovementioned reasons for choosing the range of costs applied in this study to calculate each topological measure. In two of the three measures, patients were significantly different from controls, with a lower clustering coefficient and shorter path length compared to siblings and controls. The siblings did not differ significantly from controls and did not have intermediate values in any of these measures. With regard to small‐worldness: although networks in all groups were small‐world (*σ *> 1), which may indicate that they generally had greater than random clustering but a near‐random path length, small‐worldness was not significantly different between the three groups.

The significant findings for the clustering coefficient were upheld after Simes correction (*P*_simes_ < 0.01) (Table 2).

**Table 2:** Associations between group and topological outcome measures

Average cost range 0.3–0.5	Mean (SD) of topological measures per group			Group differences on topological measures			
	Patients	Siblings	Controls	Linear trend	P vs. C	S vs. C	P vs. S
Small‐worldness	1.173 (0.097)	1.170 (0.106)	1.152 (0.096)	0.015 (0.078)	0.030 (0.075)	0.028 (0.077)	0.002 (0.876)
Clustering coefficient	0.628 (0.062)	0.649 (0.044)	0.642 (0.048)	−0.012 (0.006)^a^	−0.024 (0.006)^a^	0.003 (0.760)	−0.027 (0.001)^a^
Path length	1.662 (0.063)	1.678 (0.066)	1.674 (0.060)	−0.011 (0.047)	−0.021 (0.047)	−0.001 (0.885)	−0.020 (0.044)

SD, standard deviation; S vs. C, siblings versus controls; P vs. C, patients versus. controls; P vs. S, patients versus siblings.

Reported are *B*s and *P*‐values (in brackets). *B*s represent the regression coefficients of the multilevel regression analyses.

a Represent topological measures which are significant after Simes correction (*P*_Simes_ < 0.01).

Repeating the analyses correcting for additional confounders (tobacco, alcohol, cannabis and other drugs) or including only patients with a diagnosis of schizophrenia did not affect the pattern of results (Tables S1 and S2). Additionally, results did not change when topological measures of the patient group were compared with those of the high and low schizotypy group, that is patients had a reduced clustering coefficient compared to the high and low schizotypy group, whereas the latter two did not differ from each other (Table 3).

**Table 3:** Associations between patient‐schizotypy groups and topological measures

Average cost range 0.3–0.5	Mean (SD) of topological measures per group			Group differences on topological measures			
	Patients (*n* = 73)	HS (*n* = 72)	LS (*n* = 83)	Linear trend	P vs. LS	HS vs. LS	P vs. HS
Small‐worldness	1.173 (0.097)	1.167 (0.101)	1.158 (0.103)	0.007 (0.389)	0.015 (0.374)	0.002 (0.902)	0.013 (0.448)
Clustering coefficient	0.628 (0.062)	0.646 (0.049)	0.646 (0.044)	−0.012 (0.005)^a^	−0.025 (0.003)^a^	0.002 (0.773)	−0.027 (0.001)^a^
Path length	1.662 (0.063)	1.675 (0.059)	1.677 (0.067)	−0.009 (0.056)	−0.020 (0.046)	0.001 (0.947)	−0.021 (0.045)

SD, standard deviation; P vs. LS: patients versus; low schizotypy; HS vs. LS: high schizotypy versus low schizotypy; P vs. HS: patients versus high schizotypy.

Reported are *B*s and *P*‐values (in brackets). *B*s represent the regression coefficients of the multilevel regression analyses.

a Represent topological measures which are significant after Simes correction (*P*_Simes_ < 0.01).

No group differences were found for small‐worldness and characteristic path length after Simes correction (*P*_simes_ < 0.01) (Table 3).

### Association between topological measures and PANSS scores in patients with psychotic disorder

There was a significant negative association between the clustering coefficient and negative symptoms, although statistically imprecise by conventional alpha (clustering coefficient: *B* = −32.118, *P* = 0.017) after Simes correction (*P*_simes_ < 0.003). No associations were found for the positive, disorganized, excitement, or emotional distress PANSS symptom domains (Table 4).

**Table 4:** Associations between topological measures and psychotic symptoms

Average cost range 0.3–0.5	Positive	Negative	Disorganized	Excitement	Emotional distress
Small‐worldness	−7.178 (0.230)	−2.345 (0.786)	−5.171 (0.285)	−4.947 (0.250)	−10.199 (0.178)
Clustering coefficient	8.222 (0.401)	−32.118 (0.017)	1.853 (0.814)	−1.315 (0.853)	−0.168 (0.989)
Path length	8.920 (0.303)	−14.459 (0.240)	1.747 (0.804)	1.799 (0.774)	2.588 (0.816)

No interactions were significant after Simes correction (*P*_Simes_ < 0.003).

Reported are *B*s and *P*‐values (in brackets).

In the combined sibling and control group, there were no significant associations between topological measures and subclinical positive or negative symptoms.

### Association between topological measures and cognitive symptoms

No significant group × topological measure interactions in the models of cognitive symptoms (i.e., WM, attention, emotion processing, ToM) were found (Table 5). In the whole group, there were no significant associations between topological measures and cognitive symptoms**.**

**Table 5:** Associations between topological measures and cognitive performance and topological measures × group interactions on cognitive performance

	Main effect								Interaction							
	Arithmetic		Attention		Emotion processing		Theory of mind (ToM)		Arithmetic		Attention		Emotion processing		ToM	
	*B*	*P*	*B*	*P*	*B*	*P*	*B*	*P*	*χ* ^2^	*P*	*χ* ^2^	*P*	*χ* ^2^	*P*	*χ* ^2^	*P*
Small‐worldness	−2.90	0.244	85.96	0.155	6.76	0.320	0.66	0.614	2.16	0.340	1.26	0.534	0.08	0.961	0.28	0.868
Clustering coefficient	0.64	0.892	−131.45	0.246	−21.70	0.084	0.70	0.779	1.20	0.549	0.04	0.982	1.18	0.554	1.91	0.386
Path length	3.77	0.339	−124.50	0.174	−9.59	0.351	0.13	0.949	3.30	0.192	3.36	0.187	0.05	0.975	1.29	0.525

The *B*‐values represent the regression coefficients from multilevel random regression analysis in Stata; *P* values refer to between group differences; the χ^2^ and corresponding *P*‐values represent the results of the Wald test.

### Association between topological measures and AP medication

There was no significant association between lifetime AP use and any of the topological measures: clustering coefficient (*B* < 0.000, *P* = 0.775), path length (*B* < 0.000, *P* = 0.335), and small‐worldness (*B* < 0.000, *P* = 0.888).

## Discussion

Resting‐state functional brain networks were constructed and it was examined whether the topological properties of these networks would present possible brain (endo)phenotypes associated with psychotic disorder. Results showed that patients with psychotic disorder had a lower clustering coefficient compared to siblings and controls, which was trend‐significantly associated with negative symptoms. No significant group differences were found for path length, and small‐worldness.

### Altered topological properties in patients with psychotic disorder

The functional brain network of patients with psychotic disorder showed disturbed topological properties (i.e., lower clustering coefficient) compared to siblings and controls, which is consistent with prior fMRI and EEG studies on functional brain networks in patients with schizophrenia and healthy controls (Liu et al. 2008; Rubinov et al. 2009; Alexander‐Bloch et al. 2010; Lynall et al. 2010; Micheloyannis 2012). The clustering coefficient is a measure of functional segregation, which is the ability for specialized processing to occur within highly interconnected brain regions (Rubinov and Sporns 2010). Thus, the present results indicate that the functional network of patients with psychotic disorder has fewer functional interconnections and as a consequence is less efficient in local information transfer. Research suggests that the association of regions within clusters is highly suitable for efficient recurrent processing (Sporns et al. 2004), as well as efficient information exchange (Latora and Marchiori 2001). It is speculated that in networks of patients with psychotic disorder disrupted local information transfer may be associated with rigid communication between brain regions, clinically expressed as suboptimal functioning (e.g., reduced cognitive performance).

The characteristic path length is the most commonly used measure of functional integration, that is, the ability to quickly combine specialized information from dispersed brain regions (Rubinov and Sporns 2010). The similar characteristic path length that was found in the three groups suggests that the interactions between and across cortical regions are preserved and consequently the information transfer between brain regions is similarly fast and efficient in patients with psychotic disorder compared to siblings and controls. These results contrast previous research reporting a shorter characteristic path length (Alexander‐Bloch et al. 2010; Lynall et al. 2010) or the reverse (longer characteristic path length) (Liu et al. 2008; Yu et al. 2011) in patients with psychotic disorder compared to healthy controls. Of note, the sample size of this study (*N* = 228) was considerably higher compared to other studies (*N* < 62). Besides dissimilarities in sample size, differences in results between studies could be due to variability in the methods used to threshold the data. For example, in the studies conducted by Liu et al. (2008) and Yu et al. (2011) thresholds were chosen based on a small‐world regime, whereas Alexander‐Bloch and this study used an alternative method for thresholding the data (i.e., global thresholding based on a minimum spanning tree). In conclusion, the current evidence suggests that patients with psychotic disorder have functional network alterations although methodological differences across studies preclude definite conclusions. Therefore, using more standardized methods across symptom‐based and/or intermediate phenotypes may help to improve the level of evidence.

Contrary to our hypothesis, there were no small‐world group differences. Small‐world networks support both local specialization and global integration, possibly conferring resilience against pathological influences (Achard and Bullmore 2007). However, it has to be noted that resilience to attack (i.e., removal of nodes from the network) is associated with a heavy‐tailed degree distribution (i.e., suggesting the presence of “hubs”), which was not found in the small‐world model of Watts and Strogatz (1998). Functional integration and functional segregation are two major organizational values of the functional human brain. Once the balance between functional segregation (local specialization) and functional integration (global integration) is disturbed, small‐world organization may become more random (Rubinov and Sporns 2010). In other words, if a network has a lower average clustering coefficient and shorter characteristic path length (reduced small‐worldness) it has a higher resemblance to random networks (Watts and Strogatz 1998). Previous graph theoretical studies have shown reduced small‐world organization in patients with schizophrenia (Liu et al. 2008; Alexander‐Bloch et al. 2010). The contrast between previous and current findings may be attributed to the similar functional integration (characteristic path length) between patients and controls found in this study. It could be hypothesized that decreased small‐worldness may arise if either the increase or decrease in path length comes at the cost of a disproportionate decrease in clustering (Alexander‐Bloch et al. 2010) (representing a consequential shift in the balance between functional integration and segregation which was not noticeable in this study). However, although the small‐world measure provides insight into the organization of the functional brain network, it should not be regarded as a substitute for specific topological measures of integration (clustering coefficient) and segregation (path length) (Rubinov and Sporns 2010).

In conclusion, the present results indicate that the networks of patients with psychotic disorder are less effectively organized for local communication (i.e., reduced clustering coefficient) but with similar global communication (i.e., similar path length) as siblings and healthy controls. Thus, the reduced ability for local specialized processing only observed in the patient group may indicate state‐related network alterations.

### Topological properties in siblings of patients with psychotic disorder

Siblings of patients with psychotic disorder did not reveal similar topological alterations as patients with psychotic disorder, which is consistent with results from our previous study, using the same sample but another graph theoretical outcome measure (Peeters et al. 2015). In that study, there was no conclusive evidence for an endophenotype at the whole brain level. However, additional sensitivity analyses revealed that participants with high schizotypy scores had intermediate values between patients and the low schizotypy group at a hemispheric level (Peeters et al. 2015). A recent graph theoretical network study of patients with schizophrenia and first‐degree relatives of patients did reveal a reduced clustering coefficient in both groups with respect to controls (Lo et al. 2015). In comparison with this study, a smaller sample size (*n* = 79) was used, as well as absolute wavelet correlation matrices to construct binary undirected graphs. Although this study provided evidence that, at a whole brain level, patients (but not siblings) can be distinguished from controls, it does not exclude the possibility that topological intermediate phenotypes are more subtly distributed throughout the brain (only detectable at the level of specific brain circuits). Therefore, we are currently investigating the brain's modular community structure, which is linked to network development (Alexander‐Bloch et al. 2010), in order to examine developmental network endophenotypes of psychotic disorder (which was beyond the scope of this study).

### Association between topological measures and clinical/subclinical symptoms

To examine the effects of the investigated topological measures on behavior, this study examined the associations between the topological measures and (subclinical) psychotic/cognitive symptoms. There was no conclusive proof for associations between any of the measures and psychosis‐related symptomatology, although the association between negative symptoms and lower clustering coefficient in patients with psychotic disorder was trend‐significant after Simes correction. This may indicate that more severe negative symptoms are associated with less efficient local information transfer, either or not due to AP medication use. However, a previous graph theoretical study found an association between negative symptoms and different topological properties (i.e., characteristic path length and global efficiency) which was not observed in this study (Yu et al. 2011). These inconsistencies may be due to the analyzing techniques used by the two studies. That is, this study used Pearson correlation analysis to construct the functional connectivity matrices, whereas Yu et al. (2011) constructed functional connectivity matrices via partial correlation of ICA time courses. Also, in the latter study an uncorrected *P*‐value was used which raises concern about an elevated Type I error rate.

A possible explanation for the absence of conclusive findings may be that (subclinical) psychotic/cognitive problems are related to more specific brain circuits and therefore may not be observed at a whole brain level. Consequently, future endeavors should focus on associations between symptomatology and topological properties in specific networks or modules. Another explanation for the inconclusive findings may be that most patients were in clinical remission, as reflected by the relatively low PANSS scores with little variance.

### Methodological considerations

Particular strengths of this study are as follows: (1) the large sample size; (2) the inclusion of patients that are representative of the general population; (3) the inclusion of unaffected siblings of patients with psychotic disorder; (4) the availability of (sub)clinical symptom measures.

The average illness duration of the patients included in this study was 6.4 years and patients were in a relatively stable clinical state, which may make it difficult to generalize the present results to a more chronic or severely ill group of patients.

Most of the patients in this study were receiving second‐generation AP medication at the time of scanning. The effect of AP medication on topological network measures has, to our knowledge, only been investigated in healthy subjects (Achard and Bullmore 2007). This study acquired and analyzed fMRI data from younger volunteers (*n* = 51) and older volunteers (*n* = 11), each scanned using a resting‐state on two different occasions (placebo or sulpiride 400 mg). Results showed that dopamine antagonists reduced local and global efficiency of the network. However, in this study there was no main effect of AP on any of the topological measures.

Multiple choices are currently available for estimating the functional connectivity between brain areas, such as partial correlation, Pearson correlation, mutual information, and wavelet correlation. To our knowledge, only one study has investigated the effect of different correlation metrics on functional brain networks. They indicated that brain networks have efficient small‐world properties regardless of the correlation metric used, but significant differences exist in both global and regional topological parameters, with Pearson correlation analyses revealing the most reliable results (Liang et al. 2011). Additional studies are needed to address this question, such that future studies can use a uniform correlation metric, which would enhance the comparison between studies.

## Conflict of Interest

J. van Os is or has been an unrestricted research grant holder with, or has received financial compensation as an independent symposium speaker from, Lilly, BMS, Lundbeck, Organon, Janssen, GlaxoSmithKline, AstraZeneca, Pfizer, and Servier. M. Marcelis has received financial compensation as an independent symposium speaker from Lilly and Janssen. All other authors report no biomedical financial interests or potential conflicts of interest.

## Supporting information


**Data S1.** Topological measures.
**Table S1.** Associations between group and topological measures corrected for additional confounders
**Table S2.** Associations between group and topological measures, only including patients with schizophrenia.
