# Supplementary material for: Reduced specialized processing in psychotic disorder: a graph theoretical analysis of cerebral functional connectivity
Source: Brain Behav. 2016 Jun 29;6(9):e00508. doi: 10.1002/brb3.508 (PMC5036431; doi:10.1002/brb3.508)
Supplement: Supplementary file 1 — Data S1. Topological measures. Table S1. Associations between group and topological measures corrected for additional confounders Table S2. Associations between group and topological measures, only including patients with schizophrenia. [file BRB3-6-e00508-s001.docx]

**Supporting information**

**Topological measures**

An *N* x *N* (*N*= 90 in the present study) binary graph, *G*, consisting of nodes (brain regions) and undirected edges (functional connectivity) between nodes, is constructed by applying a threshold *T* to the Pearson correlation coefficients (Fisher’s r-to-z transformed). The subgraph *G_i_* is defined as a set of nodes that are direct neighbors of the *i-*th node, i.e., directly connected to the *i-*th node with an edge. The degree of each node *K_i,i=1,2,…,90_* is defined as the number of nodes in the subgraph *G_i_*.

This study employs the following commonly-described topological measures of networks: clustering coefficient, characteristic path length and small-worldness.

The absolute clustering coefficient of a node is the ratio of the number of existing connections to the number of all possible connections in the subgraph *G_i_*:

$$C_{i}= \frac{E_{i}}{{K_{i}\left( K_{i}-1 \right)}/2}$$

where *E_i_* is the number of edges in the subgraph *G_i_* and *K_i_* represents the degree of a node (Watts and Strogatz, 1998). The clustering coefficient is then derived by averaging the clustering coefficients of all nodes within the network:

$$C=\frac{1}{N}\sum_{i\in G} C_{i}$$

*C* is a measure of the extent of the local density or cliquishness of the network.

The mean shortest path length of a node is:

$$L_{i}=\frac{1}{N-1}\sum_{i\neq j\epsilon G} L_{i,j}$$

in which *L_i,j_* is the shortest path length between the *i-*th node and the *j-*th node and the path length is the number of edges included in the path connecting two nodes. The characteristic path length of a network is the average of the shortest path lengths between the nodes:

$$L=\frac{1}{N}\sum_{i\in G} L_{i}$$

*L* is a measure of the extent of average connectivity or overall routing efficiency of the network.

The small-worldness is defined as:

$$\sigma=\gamma/\lambda$$

with

$$\gamma=\frac{C}{C_{random}}$$

and

$$\lambda= \frac{L}{L_{random}}$$

Typically, small-world networks have similar characteristic path lengths but higher absolute clustering coefficients compared to random networks, thus, *λ* ≈ 1, *γ* > 1, and *σ* > 1 (Achard et al., 2006; He et al., 2007; Humphries et al., 2006).

To examine the small-world properties, the measures clustering coefficient and characteristic path length of the functional brain network need to be compared with those of random networks. The theoretical values of these two measures for random networks are respectively:

$$C_{random}=\frac{K}{N}$$

$$L_{random}\approx\frac{\ln\left( N \right)}{\ln\left( K \right)}$$

in which, *K* and *N* are the degree of connectivity and total number of nodes in the existing network, respectively (Achard et al., 2006; Bassett and Bullmore, 2006; Stam and Reijneveld, 2007). However, it has been suggested that making random networks with equal (or at least equal) degree as real small-world networks may not provide a valid statistical comparison (Stam et al., 2007). This is because theoretical random networks have Gaussian degree distributions which differ from the distributions of real networks being compared against (Stam et al., 2007). To obtain a robust estimate of *C_random_* and *L_random_*, we generated 100 random networks for each threshold *T* by a Markov-chain algorithm (Maslov and Sneppen, 2002; Sporns and Zwi, 2004). The values of *C* and *L* were averaged across all 100 random networks to derive a mean *C_random_* and *L_random_* for each *T*. This method has been used in previous studies (He et al., 2012; Liao et al., 2010; Liu et al., 2008; Yu et al., 2011).

**Table S1.** Associations between group and topological measures corrected for additional confounders

| **Average cost range 0.3-0.5** | **Linear trend** | **P vs. C** | **S vs. C** | **P vs. S** |
| --- | --- | --- | --- | --- |
| **Small-worldness** | 0.014 (0.152) | 0.030 (0.192) | 0.028 (0.093) | -0.002 (0.912) |
| **Clustering coefficient** | -0.014(0.007)* | -0.030 (0.003)* | 0.002 (0.809) | -0.033(0.001)* |
| **Path length** | -0.009 (0.203) | -0.018 (0.194) | 0.000 (0.966) | -0.018 (0.130) |

Reported are Bs and p-values (in brackets). Bs represent the regression coefficients of the multilevel regression analyses. S vs. C: siblings versus controls; P vs. C: patients versus. controls; P vs. S: patients versus siblings. The asterisks (*) represent topological measures which are significant after Simes correction (P_Simes_<0.01).

| **Average cost range 0.3-0.5** | **Mean (SD) of topological measures per group** | | | **Group differences on topological measures** | | | |
| --- | --- | --- | --- | --- | --- | --- | --- |
|  | **Patients (n=47)** | **Siblings (n=83)** | **Controls (n=72)** | **Linear trend** | **P vs. C** | **S vs. C** | **P vs. S** |
| **Small-worldness** | 1.163 (0.089) | 1.171 (0.106) | 1.152 (0.096) | 0.017 (0.082) | 0.030 (0.127) | 0.029 (0.058) | 0.000 (0.959) |
| **Clustering coefficient** | 0.622 (0.062) | 0.649 (0.044) | 0.642 (0.048) | -0.014 (0.006)* | -0.030 (0.002)* | 0.003 (0.749) | -0.033 (0.000)* |
| **Path length** | 1.660 (0.059) | 1.678 (0.066) | 1.674 (0.060) | -0.011 (0.088) | -0.023 (0.068) | -0.002 (0.862) | -0.022 (0.064) |

**Table S2.** Associations between group and topological measures, only including patients with schizophrenia

Reported are Bs and p-values (in brackets). Bs represent the regression coefficients of the multilevel regression analyses. SD = standard deviation; S vs. C: siblings versus controls; P vs. C: patients versus. controls; P vs. S: patients versus siblings. The asterisks (*) represent topological measures which are significant after Simes correction (P_Simes_<0.01).

**References**

Achard S, Salvador R, Whitcher B, Suckling J, Bullmore E. (2006): A resilient, low-frequency, small-world human brain functional network with highly connected association cortical hubs. J Neurosci 26(1):63-72.

Bassett DS, Bullmore E. (2006): Small-world brain networks. Neuroscientist 12(6):512-23.

He H, Sui J, Yu Q, Turner JA, Ho BC, Sponheim SR, Manoach DS, Clark VP, Calhoun VD. (2012): Altered small-world brain networks in schizophrenia patients during working memory performance. PLoS One 7(6):e38195.

He Y, Chen ZJ, Evans AC. (2007): Small-world anatomical networks in the human brain revealed by cortical thickness from MRI. Cereb Cortex 17(10):2407-19.

Humphries MD, Gurney K, Prescott TJ. (2006): The brainstem reticular formation is a small-world, not scale-free, network. Proc Biol Sci 273(1585):503-11.

Liao W, Zhang Z, Pan Z, Mantini D, Ding J, Duan X, Luo C, Lu G, Chen H. (2010): Altered functional connectivity and small-world in mesial temporal lobe epilepsy. PLoS One 5(1):e8525.

Liu Y, Liang M, Zhou Y, He Y, Hao Y, Song M, Yu C, Liu H, Liu Z, Jiang T. (2008): Disrupted small-world networks in schizophrenia. Brain 131(Pt 4):945-61.

Maslov S, Sneppen K. (2002): Specificity and stability in topology of protein networks. Science 296(5569):910-3.

Sporns O, Zwi JD. (2004): The small world of the cerebral cortex. Neuroinformatics 2(2):145-62.

Stam CJ, Jones BF, Nolte G, Breakspear M, Scheltens P. (2007): Small-world networks and functional connectivity in Alzheimer's disease. Cereb Cortex 17(1):92-9.

Stam CJ, Reijneveld JC. (2007): Graph theoretical analysis of complex networks in the brain. Nonlinear Biomed Phys 1(1):3.

Watts DJ, Strogatz SH. (1998): Collective dynamics of 'small-world' networks. Nature 393.

Yu Q, Sui J, Rachakonda S, He H, Pearlson G, Calhoun VD. (2011): Altered small-world brain networks in temporal lobe in patients with schizophrenia performing an auditory oddball task. Front Syst Neurosci 5:7.
